# Depression and anxiety symptoms among patients receiving maintenance hemodialysis: a single center cross-sectional study

**DOI:** 10.1186/s12882-022-03051-8

**Published:** 2022-12-31

**Authors:** Wei Ye, Lizhen Wang, Yu Wang, Chengjun Wang, Jingyi Zeng

**Affiliations:** 1 Department of Nephrology, Jinshan Hospital, Fudan University, Shanghai, 201508 China; 2 Department of Nephrology, Fujian Provincial Hospital, Fuzhou, 350001 China

**Keywords:** Cross-sectional study, Depression, Anxiety, Maintenance hemodialysis, Quality of life

## Abstract

**Background:**

To investigate depression and anxiety and related factors among patients receiving maintenance hemodialysis (MHD).

**Methods:**

This cross-sectional study included patients underwent MHD in 3/2022 at Jinshan Hospital affiliated to Fudan University. Depression and anxiety levels of patients were assessed using Beck Depression Inventory (BDI) and Beck Anxiety Inventory (BAI), respectively. SF-36 was used to assess patients’ quality of life. Multiple linear regression analysis was used to determine the variables associated with the scores of BDI/BAI.

**Results:**

A total of 103 patients were included, 71 cases (68.93%) and 38 cases (36.89%) with depression and anxiety, respectively. The scores of almost all domains of the SF-36 showed a declining trend with increasing depression or anxiety among patients on MHD. Higher Charlson Comorbidity Index (CCI) (β =0.066, 95%CI: 0.016-0.116, *P* = 0.010), lower educational status (β = − 0.139, 95%CI: − 0.243- -0.036, *P* = 0.009), and number of oral medications (β =0.177, 95%CI: 0.031-0.324, *P* = 0.018) were significantly associated with higher BDI scores. Longer dialysis duration (β =0.098, 95%CI: 0.003-0.193, *P* = 0.044) and number of oral medications (β =4.714, 95%CI: 1.837-7.590, *P* = 0.002) were significantly associated with higher BAI scores.

**Conclusions:**

Depression and anxiety may be likely to occur among patients undergoing MHD and impact their quality of life. Higher CCI, lower educational status and usage of multiple oral medications may be associated with depression, whereas longer dialysis duration and multiple oral medications may be associated with anxiety in MHD patients.

## Background

Depression and anxiety are the most common psychiatric conditions observed in patients receiving MHD [1, 2], with higher prevalence and incidence rates in this population than the general population [3–5]. Patients with end-stage renal disease (ESRD) face a continuous struggle with its associated symptoms and comorbidities, along with the need to cope with many stressors, including impaired biochemical and physiologic renal functions, digestive and neurologic complications, bone disease, anemia, cardiac insufficiency, decreased cognitive ability, deranged sexual function, dietary restriction as well as being functionally challenged both domestically and professionally. In addition to the underlying symptoms of the illness itself, psychological responses secondary to the chronic illness might further contribute to depression and anxiety [6–8] and in some cases lead to increased all-cause mortality [9, 10]_,_ hospitalization duration, hospitalization rates [11, 12] and inevitable socio-economic burdens [9, 13, 14]. Moreover, depression and/or anxiety were associated with poor adherence to treatment and low dialysis adequacy [15] in patients with ESRD [16].

Despite the higher prevalence of depression and anxiety among ESRD patients, these psychological conditions are usually overlooked by clinicians [17, 18]. This may be possibly due to the fact that the somatic symptoms of depression and anxiety overlap with the inherent manifestation of ESRD [19]. In general, training for nephrologists in China and, arguably, the rest of the world, lacks psychiatric and psychological aspects of patient care; as a result, their recognition of psychiatric and psychological problems is relatively low. Additionally, although selective serotonin- reuptake inhibitors (SSRIs) are the first-line medication used to treat depression and/or anxiety, its efficacy and safety among patients with chronic kidney disease (CKD) and ESRD remain uncertain [20]. Therefore, the prevention, diagnosis, and treatment of depression and anxiety in ESRD patients are still in the exploratory stage. Nevertheless, the lack of time to effectively assess and address patients’ emotional states cannot be ruled out [18].

Consequently, this necessitates the need for better understanding of the prevalence of these debilitating disorders and associated factors among patients undergoing maintenance hemodialysis. There have been several studies investigating depression and anxiety among MHD patients [21–23]. From the perspective of psychosomatic medicine, the pathogenesis of depression and anxiety is associated with physiological, psychological and social factors. Thus, this study was designed to investigate the prevalence of depression and anxiety and related factors among MHD patients and to explore the impact of depression and anxiety on their quality of life. Moreover, to screen the associated factors so as to explore new approaches for non-psychiatric clinician to cope with depression and anxiety independent of psychotropic drugs. This concept has not yet been realized and practiced by most nephrologists probably and there is also not relevant literature reported.

## Methods

### Study design and population

This cross-sectional study involved patients who were treated at The Blood Purification Center, Jinshan Hospital affiliated to Fudan University in March, 2022. Patients aged ≥18 years who did not use any prior antidepressant or anxiolytic medication and underwent hemodialysis for at least 3 months were included in this study. Patients with serious acute complications, such as acute cardiovascular and cerebrovascular events or serious infections were excluded. This study was conducted in compliance with local regulations and revised established principles of the Declaration of Helsinki (2013). The Medical Ethics Committee of Jinshan Hospital affiliated to Fudan University (Approval No: JIEC2022-S03) approved the study and written informed consent to investigation was obtained from all participants.

### Data collection and definitions

Medical records were accessed to obtain — (i) basic personal and sociodemographic information: age, gender, marital status, religious beliefs, household location, living status, parenting status (raising children), education status, and employment status; (ii) life habits: tobacco and alcohol consumption, support from social and recreational activities, and potential religious practices; travel time to dialysis facilities, (iii) clinical data: history of primary diseases, blood pressure level, duration of hemodialysis, status of kidney transplantation waiting list, number of oral medications used, vascular access failure during the previous 12 months, Charlson Comorbidity Index (CCI), (iv) laboratory examination: hemoglobin, calcium, phosphorus, parathyroid hormone, albumin, prealbumin, triglyceride, cholesterol, Kt/v, and URR (urea reduction ratio).

Comorbidities were measured using CCI. CCI is a clinical outcome-oriented scoring system comprising 16 comorbidity items with assigned weights ranging from 1 to 6, wherein higher summed scores correspond to clinical conditions of greater severity [24].

### Evaluation of quality of life

The self-administered 36-item Short Form Health Survey (SF-36) was utilized to assess the QoL in patients receiving MHD. This instrument comprises eight domains (physical functioning, role-physical, bodily pain, general health, vitality, social functioning, role- emotional and mental health). Scores of each domain can range from 0 to 100 with scores closer to 100 indicating a better QoL [25].

### Evaluation of depression and anxiety

Symptoms of depression and anxiety were assessed using Beck Depression Inventory (BDI) and Beck Anxiety Inventory (BAI) as self-administered questionnaires [26–28]. BDI and BAI consist of a 21-item self-report inventory each, designed to screen for the presence and severity of symptoms of depression and anxiety, respectively, which occurred during the previous 2 weeks. Items were scored from 0 to 3, with a total summed score ranging from 0 to 63 points. The scores indicating depression and anxiety were those greater than the cutoff of 11 and 10 points, respectively. Higher scores indicated more severe symptoms. Patients were stratified into different groups based on the following criteria: BDI < 11: no depression; 11 ≤ BDI < 20: mild depression; BDI ≥ 20: moderate/severe depression; BAI < 10: no anxiety; 10 ≤ BAI < 20: mild anxiety; BAI ≥ 20: moderate/severe anxiety [27–29]. Psychological measurement in this study adopted the method of self-report, which was completed by patients themselves independently and supervised by clinician in a quiet independent office, with a limited time of 30-40 minutes.

### Statistical analysis

All analyses were performed by the Statistical Package for Social Sciences (SPSS) version 23.0 (IBM Corp., Armonk, NY, USA) [30]. Continuous data were expressed as mean ± standard deviation (SD) and categorical data were expressed as frequency (n %). One-way ANOVA was used to compare the scores of SF-36 among patients in different groups. Multiple linear regression analysis was used to determine the variables associated with the scores of BDI/BAI. Two-sided *P* value < 0.05 was considered statistically significant.

## Results

A total of 103 patients who received MHD were included in this study. Of which, 67 (65.05%) patients were male, with mean age of 51.07 ± 11.65 years. Most of them were married (*n* = 67, 65.05%) and had children (*n* = 88, 85.44%), with 17 (16.50%) living alone. The prevalence of smoking and alcohol consumption were 25.24 and 1.94%, respectively.

The primary diseases consisted of chronic glomerulonephritis (*n* = 69, 66.99%), diabetic nephropathy (*n* = 13, 12.62%), renal vascular disease (*n* = 8, 7.76%), and others (*n* = 13, 12.62%). Patients underwent hemodialysis with a mean duration of 75.55 ± 63.77 months. The mean scores of CCI were 3.13 ± 1.82 with 15.53% of patients awaiting transplantation. Notably, the travel time to dialysis facilities for about 53.40% of patients was < 30 min, while it was more than an hour for about 27.18% of patients (Table 1).

**Table 1:** Baseline characteristics of patients receiving MHD

Variables	Value
Age (years)	51.07 ± 11.65
Gender	
Male	67 (65.05%)
Female	36 (34.95%)
Household location	
Local	90 (87.38%)
Non-local	13 (12.62%)
Marital status	
Married	67 (65.05%)
Single	36 (34.95%)
Raising children	
Yes	88 (85.44%)
No	15 (14.56%)
Living alone	
Yes	17 (16.50%)
No	86 (83.50%)
Level of Education	
Primary school or below	6 (5.83%)
Junior middle school	36 (34.95%)
Senior middle school	41 (39.81%)
University or above	20 (19.42%)
Employment status	
Yes	24 (23.30%)
No	79 (76.70%)
Religious beliefs	
Yes	10 (7.71%)
No	93 (90.29%)
Primary diseases	
Glomerulonephritis	69 (66.99%)
Diabetic nephropathy	13 (12.62%)
Renal vascular disease	8 (7.76%)
Others (e.g., LN, polycystic kidney)	13 (12.62%)
Duration of hemodialysis (months)	75.55 ± 63.77
Blood pressure level	
Systolic pressure (mmHg)	146.33 ± 24.89
Diastolic pressure (mmHg)	80.98 ± 15.23
Number of oral medications	
< 5	35 (33.98%)
5–10	56 (54.37%)
≥ 10	12 (11.65%)
Waiting list status for kidney transplantation	
Active	16 (15.53%)
Not active	87 (84.47%)
CCI (mean ± SD)	3.13 ± 1.82
Vascular access failure during the previous 12 months	
Yes	9 (8.74%)
No	94 (91.26%)
Smoking habit	
Yes	26 (25.24%)
No	77 (74.76%)
Consumption of alcohol	
Yes	2 (1.94%)
No	101 (98.06%)
Participation in social activities	
Yes	5 (4.85%)
No	98 (95.15%)
Participation in recreational activities	
Yes	38 (36.89%)
No	65 (63.11%)
Travel time to dialysis facilities.	
< 30 min	55 (53.40%)
30-60 min	20 (19.42%)
≥ 60 min	28 (27.18%)
Laboratory results	
Hemoglobin level (g/L)	108.03 ± 17.18
Serum Calcium level (mmol/L)	2.32 ± 0.30
Serum Phosphorus level (mmol/L)	2.15 ± 0.67
Parathyroid hormone level (pg/mL)	362.57 ± 293.09
Albumin (g/L)	37.92 ± 3.45
Prealbumin (g/L)	307.56 ± 78.82
Triglyceride (mmol/L)	1.90 ± 1.50
Cholesterol (mmol/L)	3.66 ± 1.18
Dialysis adequacy Kt/V	1.28 ± 0.25
URR	65.22 ± 7.07

Abbreviations: *CCI* Charlson comorbidity index, *MHD* maintenance hemodialysis, *SD* standard deviation, *URR* urea reduction ratio. Data are presented as Mean ± SD or n (%)

Of all the patients included in this study, there were 71 (68.93%) and 38 (36.89%) cases with depression and anxiety respectively, including 41 (39.81%) patients with mild depression and 30 (29.13%) with moderate/severe depression. There were 38 cases (36.89%) with anxiety out of which 23.30% (*n* = 24) had mild anxiety and 13.59% (*n* = 14) had moderate/severe anxiety. Notably, depression and anxiety coexisted in 32 (31.07%) patients (Table 2). Besides, a significant positive correlation was observed between BDI and BAI scores in patients with comorbid depression and anxiety (r = 0.444, *P* < 0.001).

**Table 2:** Depression and anxiety scores among patients on MHD

Category	BDI/BAI scores	Frequency (%)
Depression symptoms		
No	5.548 ± 3.295	32 (31.07)
Mild	14.629 ± 2.756	41 (39.81)
Moderate/ Severe	30.516 ± 7.737	30 (29.13)
Mean ± SD	16.804 ± 11.319	
Anxiety symptoms		
No	3.803 ± 2.874	65 (63.11)
Mild	13.91 ± 3.476	24 (23.30)
Moderate/ Severe	27.38 ± 9.197	14 (13.59)
Mean ± SD	9.361 ± 9.932	

Abbreviations: *MHD* maintenance hemodialysis, *BDI* Beck Depression Inventory, *BAI* Beck Anxiety Inventory. Data are presented as Mean ± SD or n (%)

Significant differences in role-physical, bodily pain, social functioning, mental health, role-emotional, general health status, and vitality (all *P* < 0.001) were found among the three graded depression groups (Table 3). Significant differences in physical functioning, role-physical, bodily pain, social functioning, mental health, role-emotional and general health status (all *P* < 0.05) were found among the three graded anxiety groups (Table 4).

**Table 3:** Comparison of QOL among the three graded depression groups

Domains	Non depression (*n* = 32)	Mild depression (*n* = 41)	Moderate/severe depression (*n* = 30)	*P* value
Physical functioning	72.17 ± 18.88	67.97 ± 21.81	60.33 ± 21.97	0.091
Role-physical	56.67 ± 39.90	47.30 ± 39.43	15.00 ± 25.09	< 0.001*
Bodily pain	82.53 ± 18.65	72.19 ± 19.82	62.72 ± 24.72	< 0.001*
General health status	50.67 ± 18.57	39.81 ± 16.60	31.33 ± 14.80	< 0.001*
Vitality	72.00 ± 13.10	59.32 ± 15.10	48.83 ± 16.01	< 0.001*
Social functioning	76.15 ± 16.83	66.76 ± 16.64	56.22 ± 20.42	< 0.001*
Role-emotional	75.56 ± 40.05	52.27 ± 44.14	21.10 ± 34.44	< 0.001*
Mental health	77.33 ± 13.43	70.92 ± 14.00	56.13 ± 16.76	< 0.001*

*P value < 0.05

**Table 4:** Comparison of QoL among the three graded anxiety groups of patients undergoing MHD

Domains	No anxiety (*n* = 65)	Mild anxiety (*n* = 24)	Moderate/severe anxiety (*n* = 14)	*P* value
Physical functioning	73.52 ± 17.16	61.90 ± 21.59	45.71 ± 23.44	< 0.001*
Role-physical	52.05 ± 40.39	19.05 ± 27.28	16.07 ± 23.22	< 0.001*
Bodily pain	79.26 ± 20.32	65.55 ± 16.93	52.86 ± 24.34	< 0.001*
General health status	44.75 ± 18.94	35.38 ± 15.15	29.29 ± 13.28	0.012*
Vitality	63.03 ± 16.72	55.48 ± 16.12	52.50 ± 19.10	0.080
Social functioning	71.41 ± 17.05	58.21 ± 19.84	54.64 ± 19.37	0.001*
Role-emotional	62.31 ± 44.09	33.30 ± 39.44	19.07 ± 38.63	0.002*
Mental health	72.46 ± 15.34	65.52 ± 19.08	54.29 ± 13.01	0.002*

Abbreviations: *MHD* maintenance hemodialysis, *QoL* quality of life. Data are presented as Mean ± SD

* *P* value < 0.05

Multivariable linear regression analysis showed that CCI (β =0.066, 95%CI: 0.016 - 0.116; *P* = 0.010), lower educational status (β = − 0.139, 95%CI: − 0.243 - -0.036; *P* = 0.009) and number of oral medications (β =0.177, 95%CI: 0.031 - 0.324; *P* = 0.018) were significantly associated with higher BDI scores (Table 5). On the other side, longer dialysis duration (β = 0.098, 95%CI: 0.003 - 0.193, *P* = 0.044) and the number of oral medications (β =4.714, 95%CI: 1.837 - 7.590; *P* = 0.002) were significantly associated with higher BAI scores (Table 6).

**Table 5:** Multivariable linear regression analysis of BDI scores among patients receiving MHD

Variables	β, 95% CI	*P* value
CCI	0.066, 0.016 - 0.116	0.010*
Lower education status	−0.139, − 0.243 - -0.036	0.009*
Number of oral medications	0.177, 0.031 - 0.324	0.018*

Abbreviations: *BDI* Beck Depression Inventory, *MHD* maintenance hemodialysis, *CCI* Charlson comorbidity index, *CI* confidence interval

* *P* value < 0.05

**Table 6:** Multivariable linear regression analysis of BAI scores among patients receiving MHD

Variables	β, 95% CI	*P* value
Dialysis duration	0.098, 0.003 - 0.193	0.044*
Number of oral medications	4.714, 1.837 - 7.590	0.002*

Abbreviations: *BAI* Beck Anxiety Inventory, *MHD* maintenance hemodialysis, *CI* confidence interval

* *P* value < 0.05

## Discussion

The study demonstrated that depression and anxiety may be likely to occur among patients undergoing MHD and impact their quality of life. Higher CCI scores, lower educational status, and multiple oral medications may be associated depression, while longer dialysis duration and multiple oral medications may be associated with anxiety.

The present study found that the overall prevalence of depression, regardless of its severity, was 68.93% among patients on MHD, which is consistent with the reports of some previous studies [31–33]. Of these patients with depression, 39.81% had mild depression symptom and 29.13% fell in the moderate to severe category. However, other studies have reported different prevalence. For instance, Othayq and Aqeeli reported that 43.6% of patients on hemodialysis experienced depression [21]. Turkistani et al. employed the Hospital Anxiety and Depression Scale (HADS) and found that approximately 23.3% Makkah patients on hemodialysis had depression [14]. Still other studies showed a higher prevalence rate of depression among hemodialysis patients. Saeed and his colleagues employed the BDI-II scoring system and observed that approximately 75% patients with ESRD on hemodialysis experienced depression [34]. Similarly, the prevalence of depression among patients in Saudi Arabia was 83% and in other parts of India was 83.5% [35, 36]. Globally, the incidence rate of depression among patients on hemodialysis ranged between 20 and 60.5% [32, 37]. As shown, there is a wide variation in the prevalence of depression across numerous studies. Different assessment tools, diagnostic criteria, customs and habits, religious beliefs, economic and cultural levels, and social security, may play roles in the different prevalence rates reported.

Among the sociodemographic and clinical factors of this patient population, higher scores of CCI, lower educational status, and number of oral medications were found to be associated with depression. The results demonstrated that patients on MHD who experienced depression presented more comorbidities, as reflected by higher scores of CCI. Our findings are further strengthened by a previous study by Brito et al., which showed that higher scores of CCI were associated with more frequent episodes of depression among MHD patients [22]. Taken together, these results suggest that the clinical presentation of depression may be highly heterogeneous and likely to be influenced by the co-presence of chronic somatic diseases.

With regard to the educational level of the patient, our findings are consistent with many previous studies of patients with ESRD [38, 39]. A study by Theofilou showed that patients with less than 9 years of education more readily experience depression than the group who had more than 9 years of education (*P* = 0.01 for severe depression) [39]. This could be possibly attributed to the discrepancy in socioeconomic status. Lower educational status not only decided the socioeconomic status and social supports obtained, but also the cognitive pattern of the disease. Moreover, the persisting physical, psychological, and economical burdens in patients undergoing MHD may lead to a chronically stressed state, and thus exacerbate depression among this patient population. In line with a previous study [21], age and gender were not correlated with depression contradicting several previous studies [40–42].

The prevalence of anxiety reported in this study was 36.89%, which is well within the reported range (1–52%) [7, 8, 43]. Further strengthening this result, a systematic review reported that the estimated prevalence of anxiety among patients on hemodialysis was 38% [44]. Of interest, our study demonstrated that longer dialysis duration was associated with greater anxiety scores in MHD patients, consistent with the findings of a previous study [45]. Further, a preliminary cross-sectional study demonstrated that MHD patients were tended to be more anxious during routine hemodialysis treatment and even minor events, such as being connected to a hemodialyzer by a non-regular nurse or on hearing the alarm of their hemodialyzer may influence their anxiety levels [1]. Behaviorism holds that anxiety is a conditioned-reflex formed by the fear of certain environmental stimuli, which is learning-dependent [29]. That is to say, anxiety can be acquired through learning. The core symptom of anxiety manifest excessive worry about some unpredictable dangerous or unfortunate events that may occur in the future. Although blood purification technology has been maturing and improved significantly, longer hemodialysis duration means more obvious chronic complications. Events such as unexpected fatal cardiovascular and cerebrovascular complications, visible body change caused by metabolic bone disease, unpredictability of the life-span of vascular access, and successive death and withdrawal of dialysis partners together psychologically influence MHD patients and lead to apprehensive expectation.

Our study found that number of oral medications were significantly associated with both depression and anxiety. To substitute some of the lost function of kidney and to address acute and chronic complications, MHD patients have to take a variety of medications orally every day, including phosphorus-binding agents; active vitamin D3; antihypertensive drugs with different mechanisms; sodium bicarbonate tablets to correct chronic metabolic acidosis; and oral medications to treat coronary heart disease, arrhythmia, and diabetes. Undoubtedly, a large number of oral medications every day and the corresponding side effects (such as gastrointestinal discomfort) can be the unavoidable daily burden faced by MHD patients, and these can become chronic stressors, leading to negative emotional responses. Therefore, as a nephrologist, it may be a good thing for MHD patients to reduce unnecessary oral medications. But this hypothesis needs further clinical study to confirm.

An interesting finding of this study was that depression and anxiety were found to be associated with almost all domains of SF-36 in MHD patients. QoL is a patients’ subjective experience of physical, psychological, and social adaptation, which reflects the state of patients’ health, prognosis, and guides clinical practices [46]. For MHD patients, inherent symptoms and treatment to the disease often have a profound impact on their QoL [47, 48]. Many studies suggested that depression and anxiety potentially affect both physical and psychological aspects of QoL [49, 50]. The present study demonstrated that, with increasing BDI and BAI scores, the levels of almost all domains of the SF-36 sharply declined, strongly suggesting that both depression and anxiety might negatively affect the physical and psychological aspects of QoL. Our findings are corroborated by a recent study that examined the rate of depression, anxiety, and the role of acceptance of their illness on patients’ QoL [43].

In this study, depression coexisted in approximately 84.2%(32/38) of patients with anxiety in MHD patients. Similar to a previous study [43], we observed a positive correlation between depression and anxiety symptoms in this study. These findings may be supported by a novel hypothesis that anxiety-depression is an inflammatory phenomenon, wherein the symptoms of both depression and anxiety are influenced by changes in different biological factors that are key to the generation of a systemic inflammatory response [51].

The present study has several limitations. First, the cross-sectional design of this study limits the generalizability of our findings as it provides information only at one time point. Second, this was a single-center study with a small sample size. Third, the symptoms of depression and anxiety were not measured at the first MHD event; therefore, time-based variations could not be evaluated. Fourth, despite using well-validated questionnaires, the occurrence of depression and anxiety among the patients was not confirmed by formal psychiatric assessments. The self-report nature of BDI/BAI can affect the results of psychological measurement according to social desirability and respondent educational attainment. In the future, additional high-quality, multi-center prospective randomized control trials employing larger patient populations are warranted.

In conclusion, depression and anxiety may be likely to occur among patients receiving MHD and may affect their quality of life. Higher CCI scores, lower educational status, and usage of multiple oral medications appear to be the independent risk factors of higher BDI scores, whereas longer dialysis duration and multiple oral medications may be independent risk factors of higher BAI scores. All above mentioned factors may probably become indicators for screening high-risk population of depression and anxiety. Subsequently, we could further explore new approaches for non-psychiatric clinician to cope with depression and anxiety independent of psychotropic drugs.

## Data Availability

All data generated or analysed during this study are included in this published article.

## Abbreviations

MHD: Maintenance hemodialysis
BDI: Beck Depression Inventory
BAI: Beck Anxiety Inventory
CCI: Charlson Comorbidity Index
ESRD: End-stage renal disease
SSRIs: Selective serotonin- reuptake inhibitors
CKD: Chronic kidney disease
SF-36: 36-item Short Form Health Survey
SPSS: Statistical Package for Social Sciences
SD: Standard deviation
